# Development of a National Agreement on Human Papillomavirus Vaccination in Japan: An Infodemiology Study

**DOI:** 10.2196/jmir.2846

**Published:** 2014-05-15

**Authors:** Haruka Nakada, Koichiro Yuji, Masaharu Tsubokura, Yukio Ohsawa, Masahiro Kami

**Affiliations:** ^1^Institute of Medical ScienceDivision of Social Communication System for Advanced Clinical ResearchThe University of TokyoTokyoJapan; ^2^School of EngineeringDepartment of Systems InnovationThe University of TokyoTokyoJapan

**Keywords:** cervical cancer, health policy, human papillomavirus, public health, vaccination

## Abstract

**Background:**

A national agreement on human papillomavirus (HPV) vaccination was achieved relatively quickly in Japan as compared to the United States and India.

**Objective:**

The objective was to identify the role of print and online media references, including references to celebrities or other informants, as factors potentially responsible for the relatively rapid national acceptance of HPV vaccination in Japan.

**Methods:**

A method of text mining was performed to select keywords, representing the context of the target documents, from articles relevant to the promotion of HPV vaccination appearing in major Japanese newspapers and Web pages between January 2009 and July 2010. The selected keywords were classified as positive, negative, or neutral, and the transition of the frequency of their appearance was analyzed.

**Results:**

The number of positive and neutral keywords appearing in newspaper articles increased sharply in early 2010 while the number of negative keywords remained low. The numbers of positive, neutral, and negative keywords appearing in Web pages increased gradually and did not significantly differ by category. Neutral keywords, such as “vaccine” and “prevention,” appeared more frequently in newspaper articles, whereas negative keywords, such as “infertility” and “side effect,” appeared more frequently in Web pages. The extraction of the positive keyword “signature campaign” suggests that vaccine beneficiaries cooperated with providers in promoting HPV vaccination.

**Conclusions:**

The rapid development of a national agreement regarding HPV vaccination in Japan may be primarily attributed to the advocacy of vaccine beneficiaries, supported by advocacy by celebrities and positive reporting by print and online media.

## Introduction

As of October 2010, 29 countries had issued formal recommendations or developed financing plans for the quadrivalent vaccine (Gardasil; Merck) and/or the bivalent vaccine (Cervarix; GlaxoSmithKline) to prevent human papillomavirus (HPV) infection [1]. Many stakeholders likely contributed to the establishment of this national agreement regarding HPV vaccination, including legislators, professional and advocacy organizations, pharmaceutical companies, print and online media, researchers, and informal networks. Of these sources, online media play an increasingly important role in the sharing of medical information [2]. Popular celebrities’ experiences have a tremendous role in raising the public awareness about this disease. In the United Kingdom, cervical cancer screenings increased after a popular TV star, Jade Goody, died from cervical cancer. This is the so-called “Jade Goody effect” [3].

Japan legally supports routine childhood immunization against only 8 diseases [4], a policy that can be primarily attributed to law suits during the 1980s and 1990s [5], and the budget deficit. Nevertheless, unlike in the United States [6] and India [7], national agreement on HPV vaccination was reached relatively smoothly in Japan. After the 2006 licensure of Merck’s HPV vaccine in the United States, bills to make HPV vaccination compulsory were introduced in 24 states. However, policymakers changed their mind and as of February 2010, only 2 states had enacted mandates [6]. In India, the government suspended the HPV vaccine trials responding to demands from advocacy groups. In contrast, in Japan, after the licensing of a bivalent HPV vaccine in October 2009, 294 local governments corresponding to 16.4% of the total local governments decided to offer subsidies by October 26, 2010. On August 5, 2010, the Minister of Health, Labor and Welfare proclaimed that they would budget for the national vaccination for the next fiscal year. These actions by the government imply that a national agreement for HPV vaccination was reached. The objective of this study is to identify the role of print and online media references, including references to celebrities or other informants, as factors potentially responsible for the relatively rapid development of the national agreement on HPV vaccination in Japan.

## Methods

### Overview

**Figure 1:**
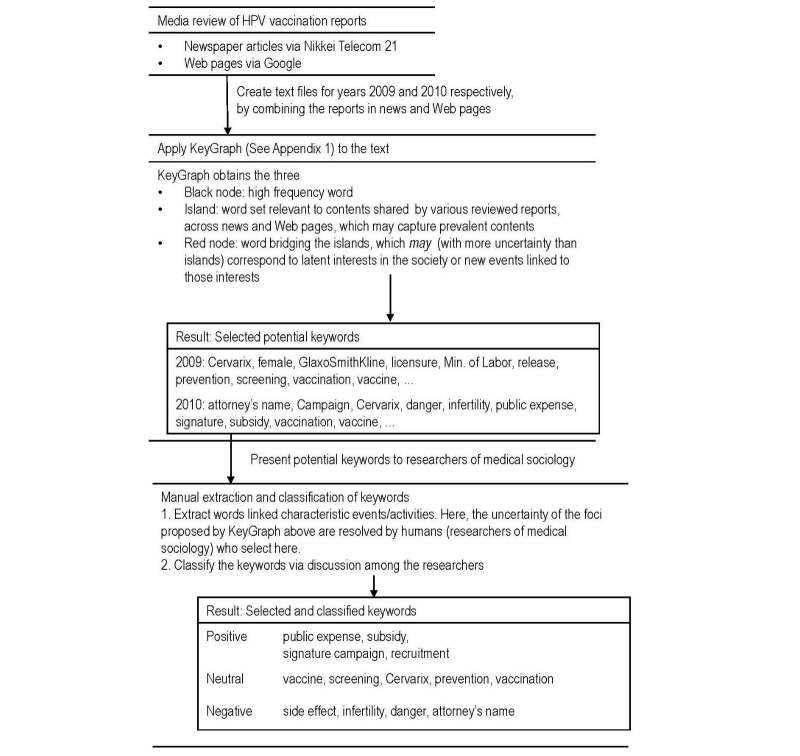
Overview of the process to analyze media review reports on HPV in Japan.

An overview of the process and the analysis method used in this paper is shown in Figure 1. We will describe the details of each step in the process, corresponding to each frame in Figure 1, subsequently.

### Media Review

After review of articles related to cervical cancer using keywords, a total of 5 newspapers covering approximately 60% of total circulation were selected from among all registered newspapers in Nikkei Telecom 21, the major newspaper database in Japan. Using the Google search engine, we selected Web pages by keywords and the names of 5 Japanese celebrities who survived cervical cancer before 2010; most of these celebrities were involved in activities to promote HPV vaccination or had shared their stories via books or mass media. The monthly change in the number of instances in which each category of keywords appeared in newspaper articles and Web pages was determined and analyzed. We collected Web pages updated for specified periods via Google’s search engine. The dates of publication for news articles were listed in the database. All articles and almost all the Web pages were in Japanese because we searched using Japanese keywords (the keywords shown subsequently are translated into English). As we described the situation in Japan, the language was constrainted to Japanese.

### Keyword Selection and Classification

Keywords were selected using KeyGraph [8-13] (see Multimedia Appendix 1 for details) and were manually classified into positive, negative, or neutral expressions so that their frequency in newspaper articles and Web pages between January 2009 and July 2010 could be compared.

**Figure 2:**
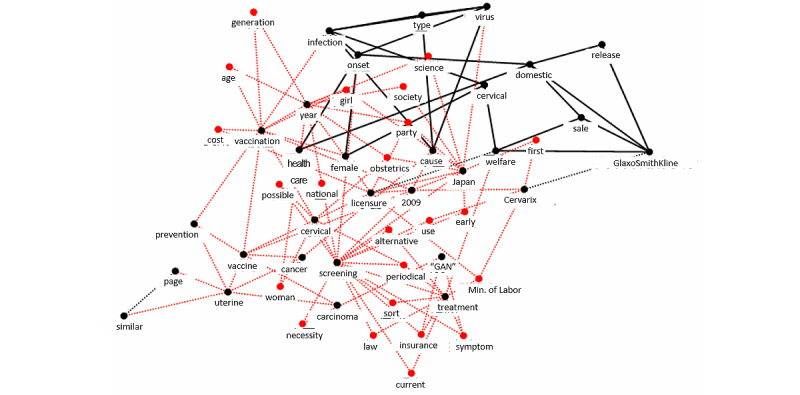
Keyword map (KeyGraph) showing the primary stakeholders in HPV vaccination in 2009. The most frequent words are shown with black nodespairs of these frequent words co-occurring the most get linked via black lines. “GAN” is a Japanese word meaning cancer.

**Figure 3:**
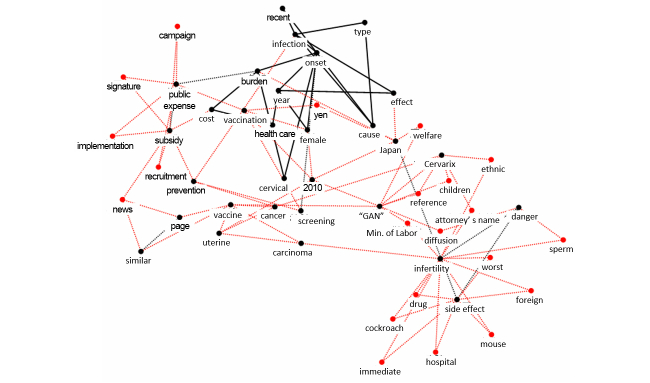
Keyword map (KeyGraph) showing the primary stakeholders in HPV vaccination in 2010. The most frequent words are shown with black nodespairs of these frequent words co-occurring the most get linked via black lines. “GAN” is a Japanese word meaning cancer.

We used the keyword graphs in Figures 2 and 3 obtained by KeyGraph applied to the review of the reporting of HPV vaccination during the study period. Referring to Multimedia Appendix 1, the number of nodes (element of the document) were set as N_blacknodes=30, N_black links =20, and N_red nodes=10 (the default values for setting these parameters). The black node, red node, and black link are defined as a high frequency word, an important word not shown with high frequency in the document, and a connection in the document, respectively.

From the keywords presented by KeyGraph, 2 domain experts, who are researchers of medical sociology (HN and MK), independently selected words representing already existing concepts relevant to the promotion of HPV vaccine and classified the keywords as positive, neutral, or negative (Table 1), based on discussion with specialists in vaccination policy in Japan. If there was discordance between them, HN made a final decision. The kappa statistic was used to assess the level of agreement among the researchers. The definition of positive, negative, and neutral were “promotion of HPV vaccination,” “objection or barrier to HPV vaccination,” and “activities against cervical cancer,” respectively. For example, “public expense” was positive because HPV vaccination was supported by public funding. An attorney’s name was negative because the attorney actively developed a campaign against HPV vaccination.

**Table 1:** Classification of keywords associated with human papillomavirus (HPV) in Japan.

Category	Keywords
Positive	Public expense, subsidy, signature campaign, recruitment
Neutral	Vaccine, screening, Cervarix, prevention, vaccination
Negative	Side effect, infertility, danger, [an attorney’s name]

## Results

### Media Analysis

**Figure 4:**
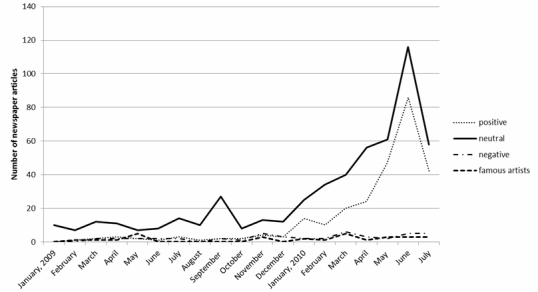
Serial changes in the number of newspaper articles containing positive, negative, and neutral keywords related to cervical cancer and the names of relevant Japanese celebrities.

**Figure 5:**
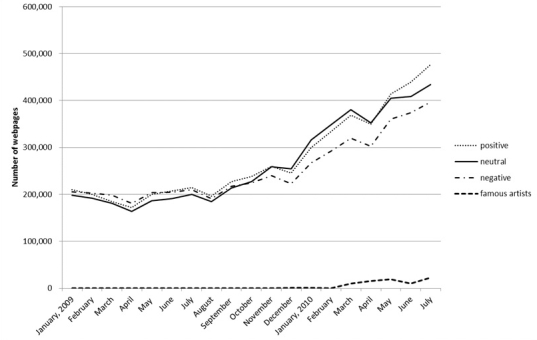
Serial changes in the number of Web pages containing positive, negative, and neutral keywords related to cervical cancer and the names of relevant Japanese celebrities.

The total number of newspaper articles and Web pages containing information regarding cervical cancer was 624 and 15,792,600, respectively, for the study period. An increase in the number of newspaper articles coincided with an increase in Web pages in early 2010 (Figures 4 and 5). The numbers of positive and neutral keywords in the newspaper articles increased sharply in early 2010 and peaked in June 2010, whereas the number of negative keywords remained low throughout the study period (Figure 4). In contrast, the numbers of positive, neutral, and negative keywords in Web pages increased gradually over the study period and did not significantly differ by category (Figure 5). Although the number of Web pages containing the names of relevant Japanese celebrities had increased to 19,200 by May 2010 (Figure 4), few newspaper articles reported on these celebrities during the study period (Figure 5). The neutral keywords “vaccine” and “prevention” appeared more frequently in newspapers, whereas the negative keywords “infertility” and “vaccine” appeared more frequently on Web pages.

### Keyword Analysis

The assessment of the agreement on the keyword labeling revealed no significant differences between the 2 researchers (κ=.884). The number of Web pages containing the 3 positive keywords increased gradually over the study period, whereas the number of newspaper articles containing the positive keywords “subsidy” and “public expense” peaked in June 2010. The neutral keywords “vaccine” and “prevention” co-occurred frequently in newspaper articles throughout the study period, with the number containing “prevention” peaking in June 2010. Although the number of Web pages containing the negative keywords “side effect” and “infertility” increased gradually over the study period, the number of newspaper articles containing “side effect” peaked in June 2010.

## Discussion

### Principal Findings

Text mining using KeyGraph led to extraction of the keywords “public expense,” “subsidy,” and “signature campaign,” but not “pharmaceutical company” (a main stakeholder in the promotion of HPV vaccination [14-16]) as a positive keyword. The extraction of these words—particularly “signature campaign,” a type of campaign conducted by vaccine beneficiaries—suggests that vaccine beneficiaries cooperated with providers in promoting HPV vaccination in Japan. Because previous studies on HPV vaccination failed to find evidence of such cooperation [17], these findings were unexpected but noteworthy in discussing latent social trends affecting the prevalence of HPV vaccination.

Analysis of the results suggested that the most influential stakeholders had changed from vaccine providers, including medical specialists and pharmaceutical companies, to vaccine beneficiaries during the nationwide discussion on HPV vaccination between 2009 and 2010 (Figures 2 and 3). After comparing Figures 2 and 3, we defined stakeholders as people or groups who might have some impact or influence on the HPV vaccination situation in Japan. The words describing HPV, features of cervical cancer, vaccine, or the name of the pharmaceutical company appeared with high frequency (Figure 2). The media primarily reported the development or licensure of HPV vaccine in 2009, implying that the main stakeholders were vaccine providers. In contrast, the keywords “public expense,” “subsidy,” “signature campaign,” and an attorney’s name appeared in Figure 3. These keywords were closely related to lay people’s activities or financial issues related to individual vaccination. The increase in the appearance of the keywords implies that the stakeholders in 2010 were lay people who generally benefit from vaccine.

The nature of the discussion regarding HPV vaccination varied widely between newspaper articles and Web pages. We found 2 major tendencies: (1) the number of newspaper articles containing positive and neutral keywords increased exponentially before peaking in June 2010, but the number containing negative keywords remained stable (Figure 4), and (2) the number of Web pages containing all 3 categories of keywords increased gradually over the study period (Figure 5). One possible explanation for this difference is timing. When the Japanese government initiates budgetary compilations, typically in May or June, it allows press clubs—including the major newspaper and television providers, but not online media sources—a high level of access to its proceedings [18]. Thus, the nature of reporting by print and online sources may differ during these periods. Another possible factor is bias. Newspapers are often financially dependent on their sponsors, which may include pharmaceutical companies [19]; thus, they may be reluctant to report information opposing their sponsors’ interests. Another possible factor may be that print media have strict space and time restrictions. Because of these restrictions, newspaper media sometimes report only one aspect of the events.

In contrast, anyone can provide online content, and many of these authors have no conflicts of interest with sponsors. Web content writers, especially amateur writers, can provide their opinions freely on Web. Moreover, because Web content remains almost permanently, they are gradually accumulated.

**Figure 6:**
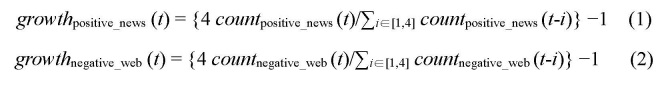
Equations for each month (t) where growth x(t) denotes the increase in the counted number, countx(t) of articles or Web pages corresponding to x at time t, in comparison with the average of the 4 preceding months.

This discussion does not prove causality, but proposes hypotheses consistent with the observed trends. Another possibility is that, for example, regardless of superficial differences regarding words and causations, the 2 tendencies are interrelated. Their coupling effect can be suggested as a hypothesis, according to the following analysis. The curve in Figure 4 for newspaper articles, in which positive expressions correspond to persuasive messages may superficially look different from the curve in Figure 5, in which negative expressions (ie, criticisms and counter opinions to HPV vaccination) persistently increased on the Web. However, we found a significant covariation between the 2 tendencies. We calculated the values from the equations displayed in Figure 6 to see correlation between the 2 tendencies.

**Figure 7:**
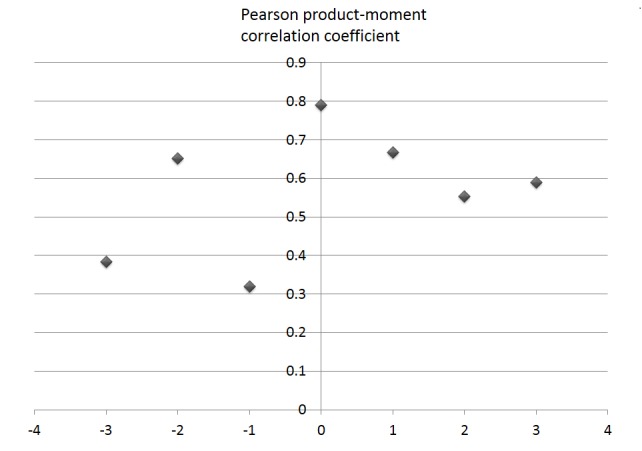
Pearson correlation coefficient between the 2 sequences corresponding to positive keywords in news and negative keywords on the Web, with staggering the sequences by sliding time dt.

For example, growth of positive news (May 2010) is equal to 30/17, because the value of count positive news (May 2010)—the number of articles with positive words in May 2010, equal to 47 as shown in Figure 3—is larger than 17 by 30, which is the average of count of positive news for the 4 months from January to April 2010. We used 4 months as the denominator in both equations to omit noise that appeared as small variations over periods of 4 months. Then, we extinguished the outlying period January 2010, where growth of positive news (January 2010) was 4.09. This value was exceptionally large in comparison with other periods, where the second largest growth of positive news was 2.4 (SD 1.18). As a result, as in Figure 7, the sequences of growth of positive news (*t*) and growth of negative Web (*t*-dt) are correlated significantly for dt=0, where dt means the length of staggered time in evaluating the temporal correlation between the sequences. That is, the strong correlation (*R*=.79) for dt=0 means the coincidence of changes in the frequencies of words in each category (positive/neutral/negative), between the 2 sequences for exactly same periods (May 2009 to June 2010). Thus, the negative reaction of the Web can be considered to be linked significantly with the positive in the mass media represented by newspapers.

One of the potential explanations for the tendencies is the 2-sided presentation, although its causal relation to the national agreement about HPV vaccination in Japan is difficult to prove until we observe sufficient variation of situations (ie, whether each piece of candidate causal information was shown or not for each change of national agreement—this point is addressed in future work mentioned subsequently). This effect, established by Hovland et al [20], was that a 2-sided message—a message presenting not only the negative side but also the positive side of a controversial issue (such as an advertised product)—is more persuasive than a 1-sided message to educate an audience expected to have basic knowledge about the issue discussed or to those who were not initially positive to be persuaded by a message. This difference between the effects of 1- and 2-sided messages is explained in terms of perception of bias in Belch [21]. That is, a 1-sided message would be regarded as biased by those who are aware of opposing arguments or of arguments on both sides of the issue. Taking this position, the analysis result presented here corresponds to this effect in that the positive information provided by newspapers and the negative information on the Web composed 2-sided messages and were casted before and with the social movement toward the promotion of vaccination.

### Limitations

We have tried to present a plausible explanation for the media trends and national agreement on HPV vaccination. We should consider 2 limitations faced by this study that may limit the generalizability of the findings. First, certain types of media, including magazines, television, and social media (ie, Twitter and Facebook)—the last of which have become increasingly important sources of information transmission between doctors and patients as well as among medical researchers [19,22]—were not included in the analysis because of their lack of inclusion in major databases. Because we do not have a well-established database of social media content, we focused on newspapers and Web pages in this study. Further investigation of social media will be necessary in our next study. Second, pharmaceutical companies might have indirectly influenced the national agreement by promoting HPV vaccination through donations to patient advocacy groups or nonprofit organizations. Because no database was available about advertisements in newspapers and on Web media in Japan, it is difficult to evaluate its influences on the public opinion. Nagata and colleagues [23] reported the effects of advertisements in weekly magazines. According to the study, 6 weekly magazines provided 696 articles and 340 advertisements relating to cancer, 30.4% of which reported dubious folk medicine and immunotherapeutic treatments without supporting evidence. The contents in the weekly magazines could prejudice the public against cancer therapies. Pharmaceutical companies may intend to influence public opinion via mass media. Both the first and the second limitations are addressed in our future work identifying the role of references to information as factors responsible for the development of national agreement on HPV vaccination. We expect this will present plausible explanations for the positive social trend and support professions in the future who would contribute to prevailing useful medical technologies.

### Conclusion

The rapid development of a national agreement regarding HPV vaccination in Japan may be primarily attributed to advocacy by vaccine beneficiaries supported by celebrity advocacy and positive reporting by print and online media.

## Multimedia Appendix 1

KeyGraph is visualizing a word map for keyword extraction.

## Abbreviations

dt: length of staggered time evaluating temporal correlation between sequences
HPV: human papillomavirus
