# Supplementary material for: Development of a National Agreement on Human Papillomavirus Vaccination in Japan: An Infodemiology Study
Source: J Med Internet Res. 2014 May 15;16(5):e129. doi: 10.2196/jmir.2846 (PMC4051745; doi:10.2196/jmir.2846)

Appendix

KeyGraph®: Graphical data mining for Extracting Keywords

KeyGraph(R)[1, 2, 3, 4, 5] (trademark registered by Yukio Ohsawa), a tool for visualizing a map of correlations among frequent and infrequent words in a document has been applied to documents collected.

If applied to text (a document), KeyGraph's feature is to enable to show infrequent words that may represent the context of the document but do not outstand as far as one highlights words that are frequent or relatively frequent than in other documents words.

As a result, the extracted keywords reflect the latent context such as emotion[3], team-work motivation[4], and causes of communication gaps [5], that are not easy to capture in other indexing methods.

KeyGraph is composed as follows (See also a Figure): In the first step, a certain (given) number ($N_{blacknode}$) of the most frequent words are extracted from the target text, and shown with black nodes. Then in Step 2, a certain number ($N_{blacklink}$) of pairs of these frequent words co-occurring the most (the extent of this co-occurrence is computed here using Jaccard coefficient as in the literature[2], which means the percentage of sentences including both of the pair among all sentences including either of the pair) get linked via black lines. Each connected graph obtained hereby forms one *island* -- a word set appearing in various parts of the text and implying a context shared by those parts. Such a context shared widely tends to be a common sense or a basic concept in the domain of documents. Third, candidates of keywords including a given number ($N_{rednode}$) of red nodes are obtained. Here, all words are sorted by the probabilities to occur in sentences including words from two or more islands -- the highly ranked nodes in this measure can be regarded as bridges among islands. And, the words from the top in this list to the last (i.e., $N_{rednode}$ -th) red node are given as keyword candidates.

Simply put with the resultant graphs, e.g., Figure 1 and 2, all the red nodes, and black nodes connected to multiple red (weakly colored than black ones in the grayscale print version) nodes are given as candidates of keywords, because they are supposed to contribute to the connections of common senses and prevalent concepts. We assume the context of the social trend, underlying presented documents, causes news and opinions via making these connections. And, it is humans (researchers of medical sociology in this paper) that finally select and classify keywords among these candidates, because they know latent contexts learned from active daily life in the real society.

The default number of nodes are set as $N_{blacknodes}$ = 30, $N_{blacklink}$ = 20, and $N_{rednode}$ = 10, according to authors' and users' experiences. Note here that, if multiple equally ranked nodes exist at the border, the obtained nodes and links will be set to more or less than these given values. For example, if there are three 30th most frequent words in the target text, we obtain 32 black nodes by KeyGraph. On the other hand, if one hundred 30th most frequent words are obtained, they are discarded from the

procedure and the 29th most frequent word remains.

References in Appendix 1

1. Ohsawa, Y., Benson, NE., and Yachida, M., KeyGraph: Automatic Indexing by Co-occurrence Graph based on Building Construction Metaphor, Proc. Advanced Digital Library Conference (IEEE ADL'98) 1998; 12-18. DOI: 10.1109/ADL.1998.670375.
2. Ohsawa, Y., and McBurney, P., Chance Discovery, Springer NY (2003)
3. Takita, M., Tanaka, Y., Kodama, Y., Murashige, N., Hatanaka, N., Kishi, Y., Matsumura, T., Ohsawa, Y., and Kami, M., Data mining of mental health issues of non-bone marrow donor siblings, Journal of Clinical Bioinformatics 2011, 1:19. PMID: 21884635.
4. Fruchter, R., Ohsawa, Y., and Matsumura, N.,: Knowledge Reuse through Chance Discovery from an Enterprise Design-Build Enterprise Data Store, New Mathematics and Natural Computation, 1(3):393--406, 2005. DOI: 10.1142/S1793005705000263.
5. Abe, A., Ohsawa, Y., Ozaku, H.I., Sagara,K., Kuwahara, N., and Kogure, K.: Communication Error Determination System for Multi-layered or Chained Situations. Fundamenta Informaticae, 98(1):123-142, 2010. DOI: 10.3233/FI-2010-220.

Figure: The two steps of KeyGraph®

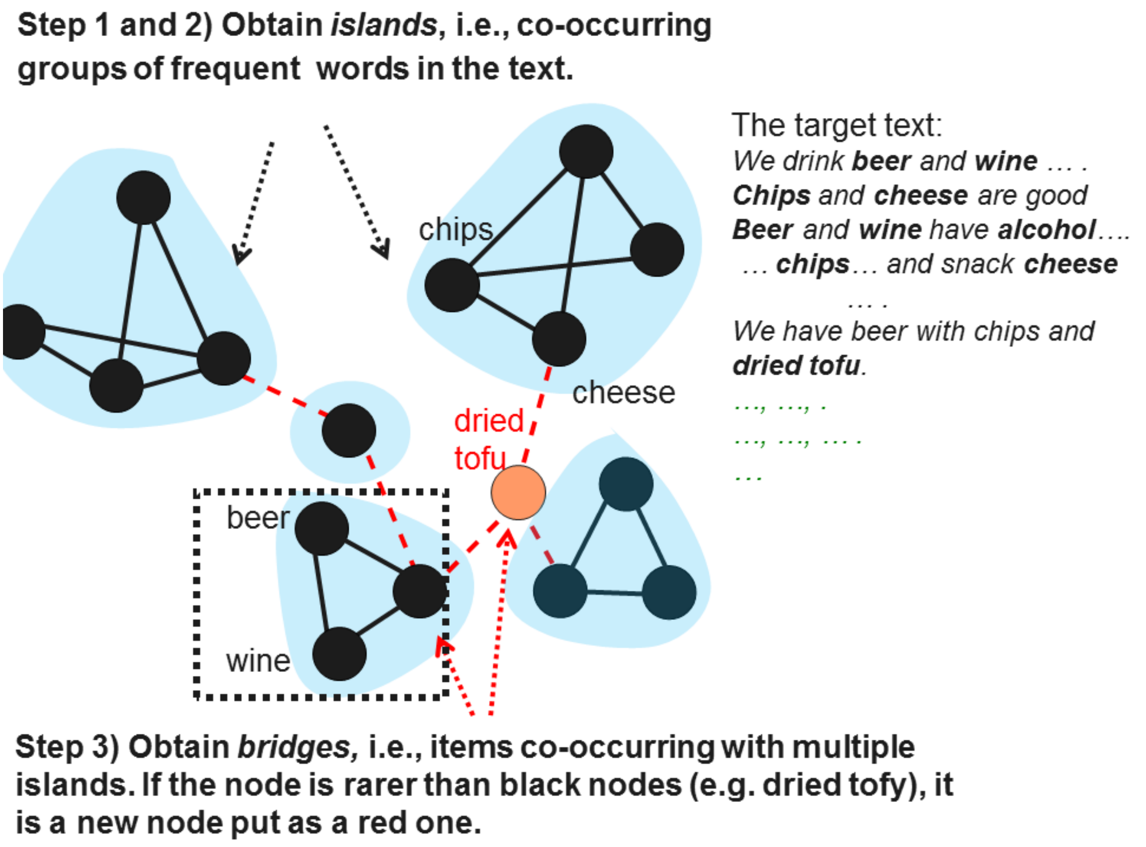

Supplement: Supplementary file 1 [file jmir_v16i5e129_app1.pdf]
